# Supplementary material for: JNK regulates muscle remodeling via myostatin/SMAD inhibition
Source: Nat Commun. 2018 Aug 2;9:3030. doi: 10.1038/s41467-018-05439-3 (PMC6072737; doi:10.1038/s41467-018-05439-3)
Supplement: Supplementary file 1 — Supplementary Information [file 41467_2018_5439_MOESM1_ESM.pdf]

Supplementary Information File:

JNK regulates muscle remodeling via Myostatin/SMAD inhibiton

Lessard et al., *Nature Communications*, 2018

A Voluntary Wheel Running

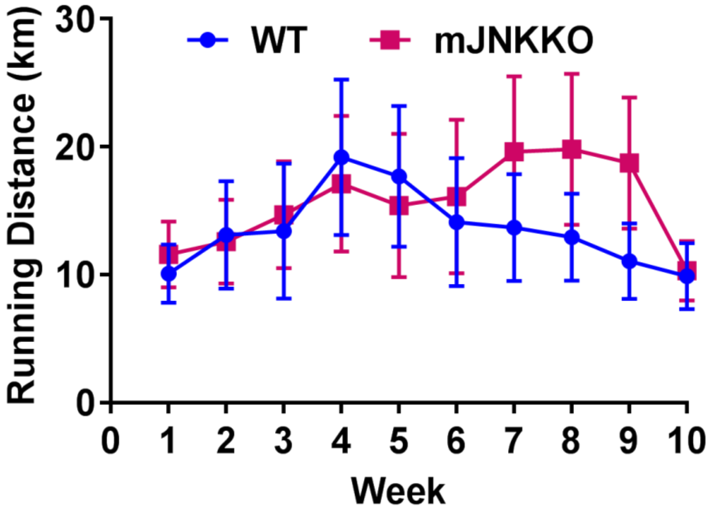

B Cumulative Running Distance

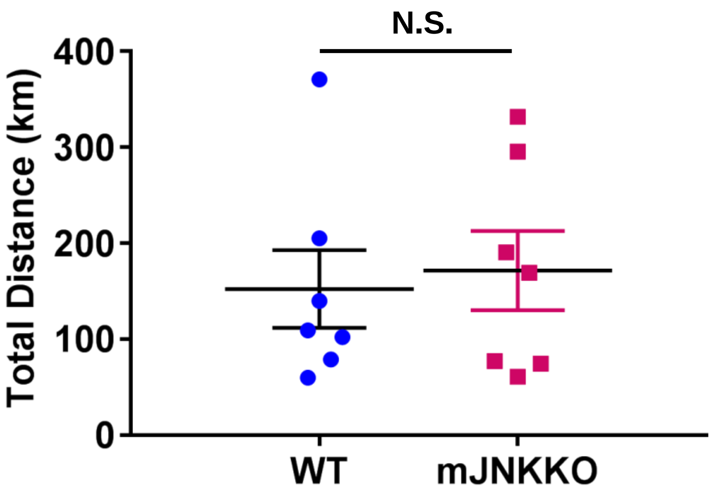

Supplementary Figure 1. Muscle-specific JNK1/2 knockout mJNKKO mice and wild-type (WT) were housed in cages with running wheels for 10 wks. A) Average weekly voluntary running distance, and B) Total running distance were calculated. N=7 animals/group. Data are presented as Mean +/- SEM. N.S., not statistically significant by unpaired T-Test.

Supplementary Figure 2
Uncropped Blots from Figure 3A

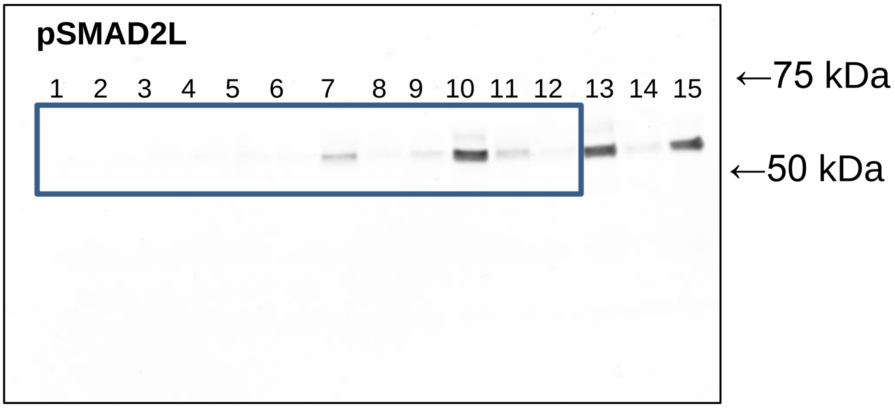

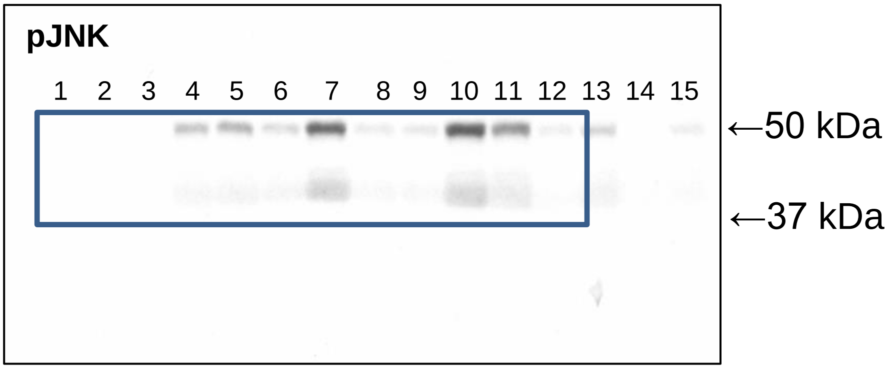

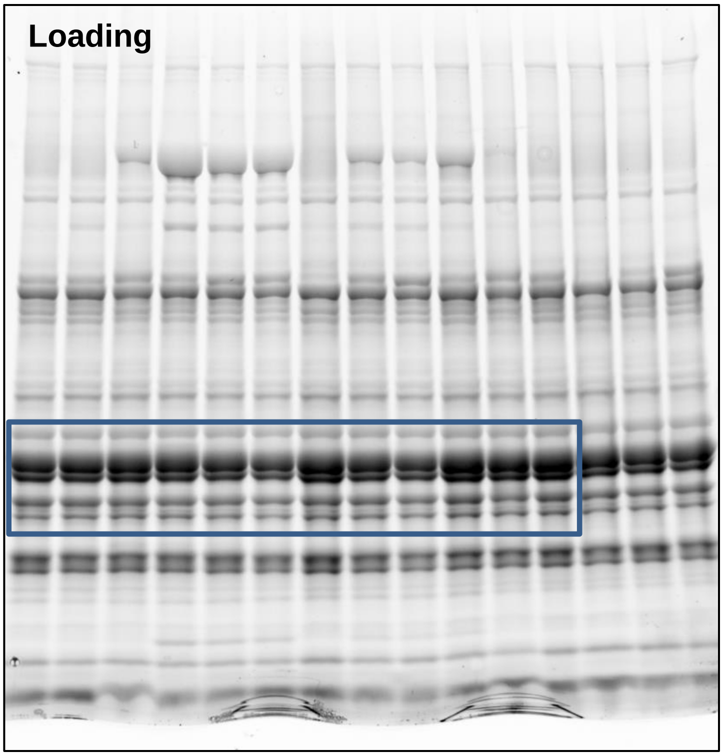

Mouse Treadmill Running
Lanes:
1-3: Rest
4-6: 15 min running
7-9: 30 min running
10-12: 60 min running
13-15: 60 min running + 30 min rest

Uncropped Blots from Figure 3B

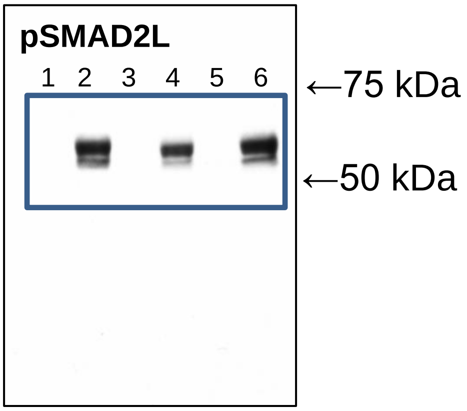

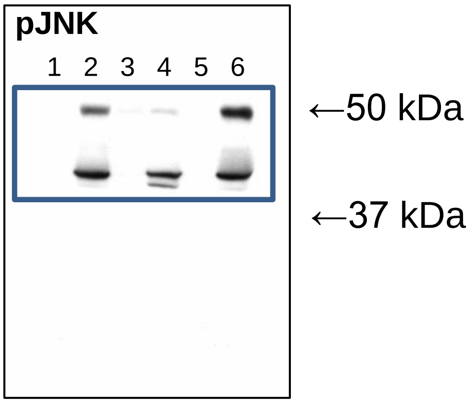

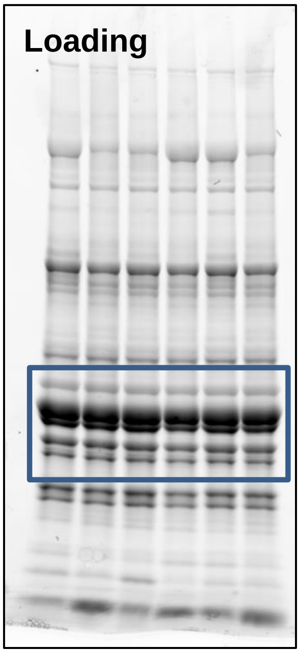

Mouse In Situ Contraction

Lane:

1: Mouse 1 Basal leg

2: Mouse 1 Contracted leg

3: Mouse 2 Basal leg

4: Mouse 2 Contracted leg

5: Mouse 3 Basal leg

6: Mouse 3 Contracted leg

Uncropped Blots from Figure 3C

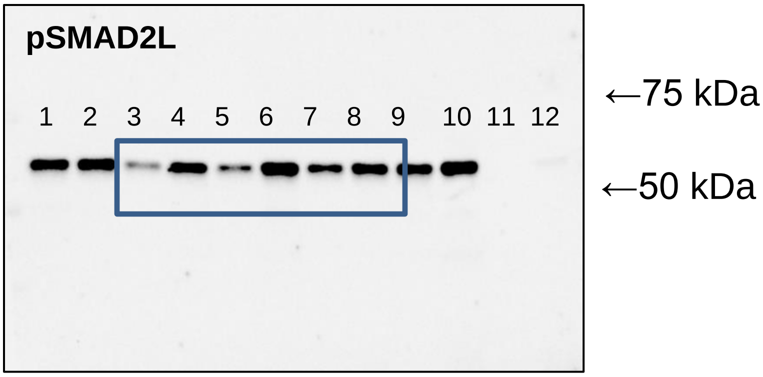

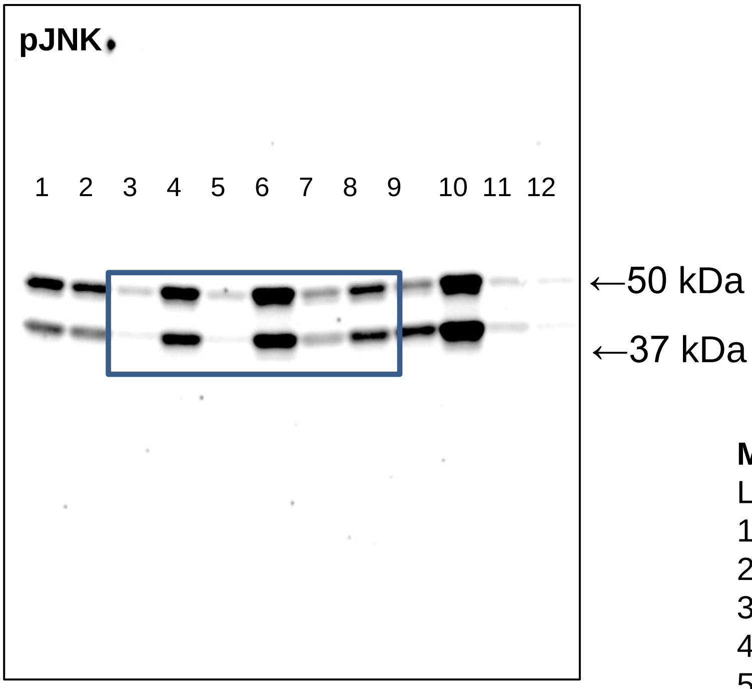

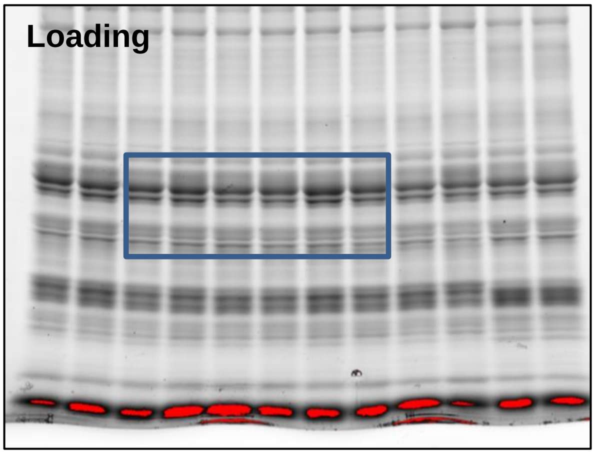

Mouse Stretch Experiments
Lane:
1: Mouse 1 Basal Muscle
2: Mouse 1 Stretched Muscle
3: Mouse 2 Basal Muscle
4: Mouse 2 Stretched Muscle
5: Mouse 3 Basal Muscle
6: Mouse 3 Stretched Muscle
7: Mouse 4 Basal Muscle
8: Mouse 4 Stretched Muscle
9: Mouse 5 Basal Muscle
10: Mouse 5 Stretched Muscle
11: Mouse 6 Control Muscle
(flash frozen: Non-incubated)
12: Mouse 6 Control Muscle
(flash frozen: Non-incubated)

Uncropped Blots from Figure 4A

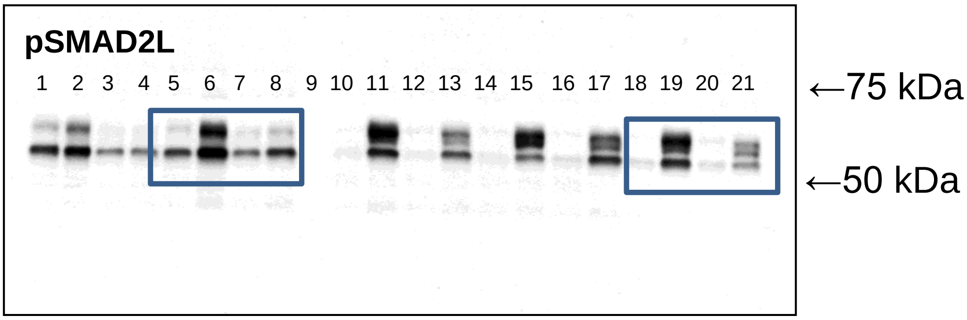

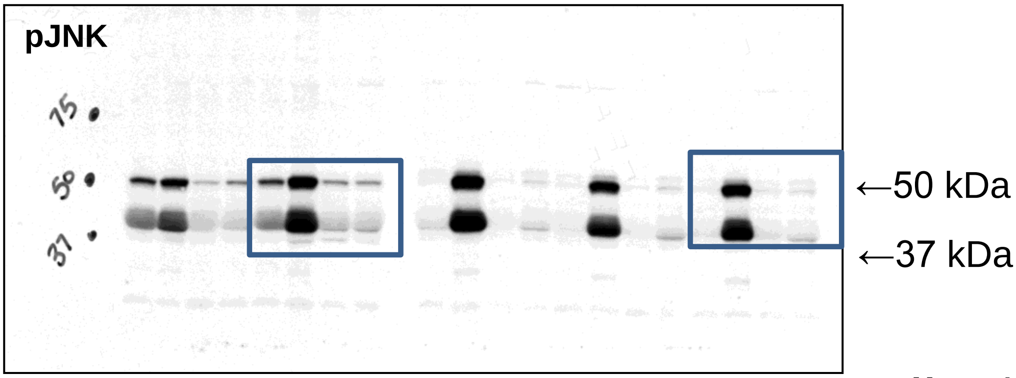

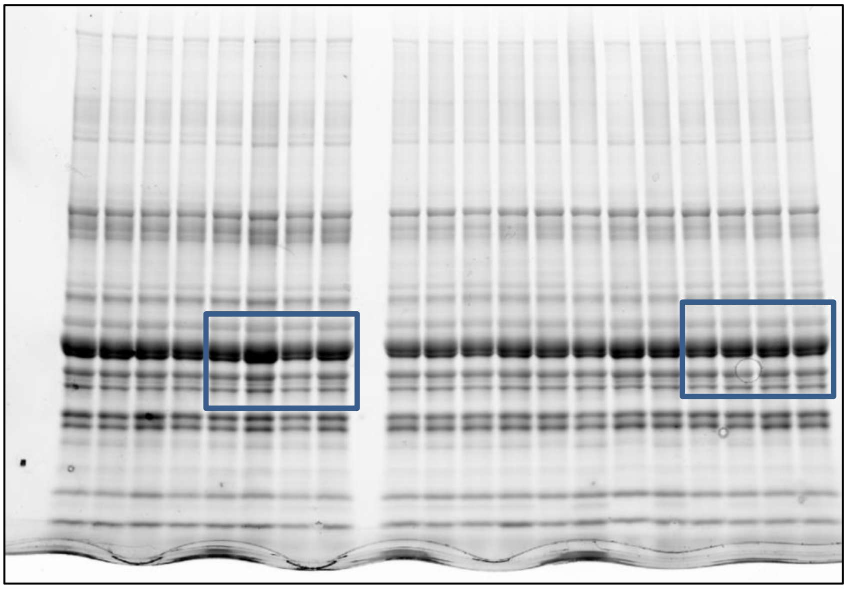

Mouse In Vitro Contract Experiment
Lane:
1: WT 1 Basal Muscle
2: WT 1 Contracted Muscle
3: KO 2 Basal Muscle
4: KO 2 Contracted Muscle
5: WT 3 Basal Muscle
6: WT 3 Contracted Muscle
7: KO 4 Basal Muscle
8: KO 4 Contracted Muscle
9: Empty Lane

Mouse In Situ Contract Experiment Lanes:
10: WT 5 Basal Muscle
11: WT 5 Contracted Muscle
12: KO 6 Basal Muscle
13: KO 6 Contracted Muscle
14: WT 7 Basal Muscle
15: WT 7 Contracted Muscle
16: KO 8 Basal Muscle
17: KO 8 Contracted Muscle
18: WT 9 Basal Muscle
19: WT 9 Contracted Muscle
20: KO 10 Basal Muscle
21: KO 10 Contracted Muscle

WT: Wild-type Control Mouse
KO: mJNKKO Mouse

Uncropped Blots:
Figure 4D

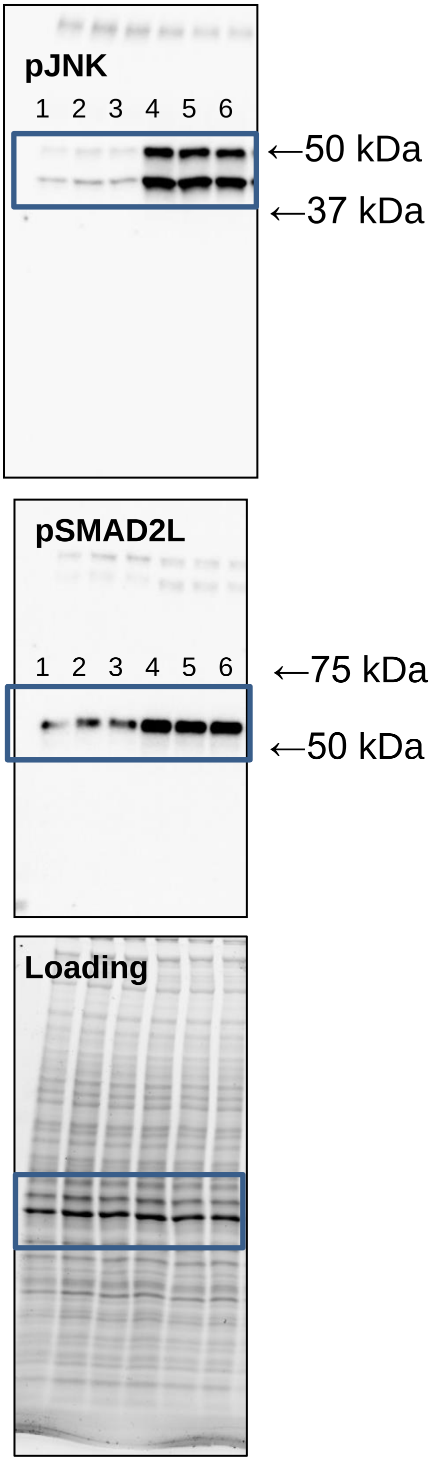

C2C12 Myoblasts
Lanes:
1-3: Vehicle
4-6: 5 µM Anisomycin

Uncropped Blots:
Figure 4E

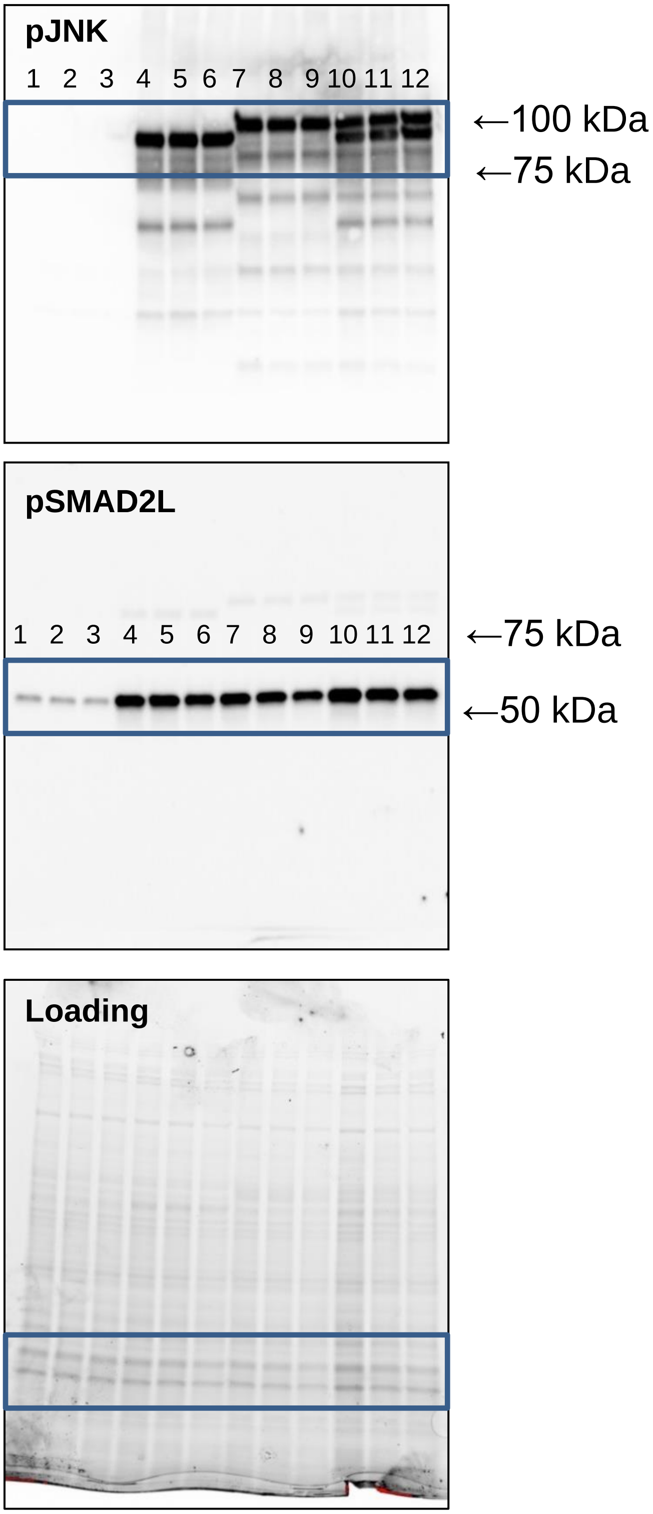

C2C12 Myoblasts
Lanes:
1-3: Empty Vector
4-6: Active JNK1 overexpression
7-9: Active JNK 2 overexpression
10-12: Active JNK1 and JNK2 overexpression

Uncropped Blots from Figure 4F

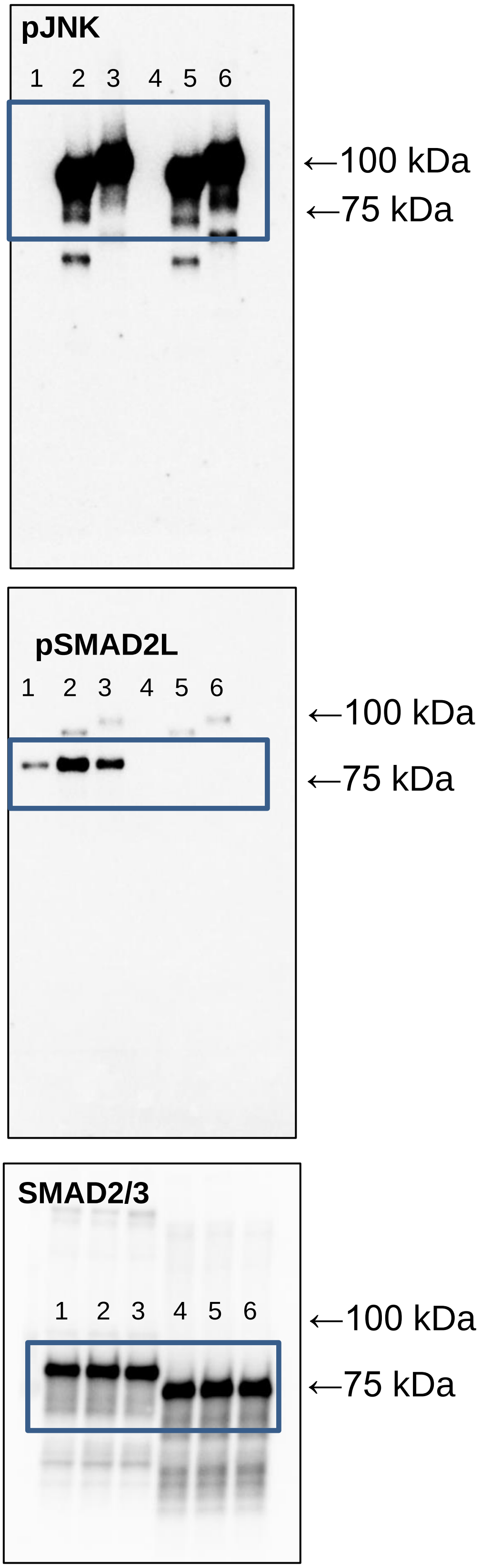

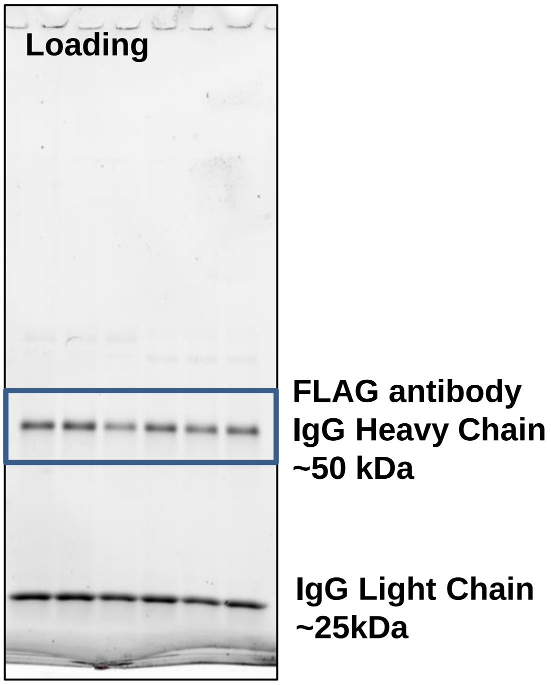

In vitro JNK activity assay experiment

Lane

1: Empty Vector + Recombinant SMAD2

2: Active JNK1 + Recombinant SMAD2

3: Active JNK2 + Recombinant SMAD2

4: Empty Vector + Recombinant SMAD3

5: Active JNK1 + Recombinant SMAD3

6: Active JNK2 + Recombinant SMAD3

Uncropped Blots from Figure 5B

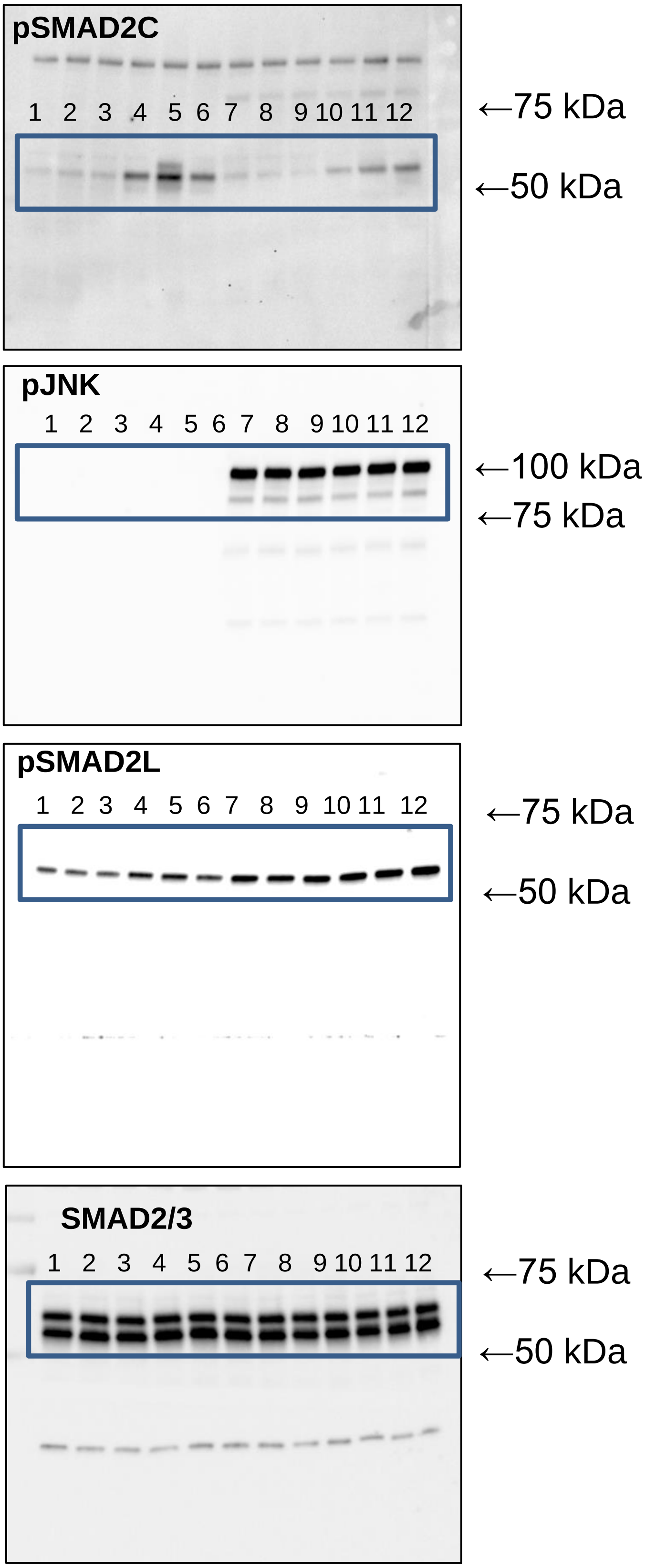

Active JNK + Myostatin Experiment
Whole-Cell Lysates

Lane
1-3: EV + Vehicle Control
4-6: EV + 20 nM Myostatin
7-9: Active JNK + Vehicle Control
10-12: Active JNK + 20 nM Myostatin

Uncropped Blots from Figure 5C

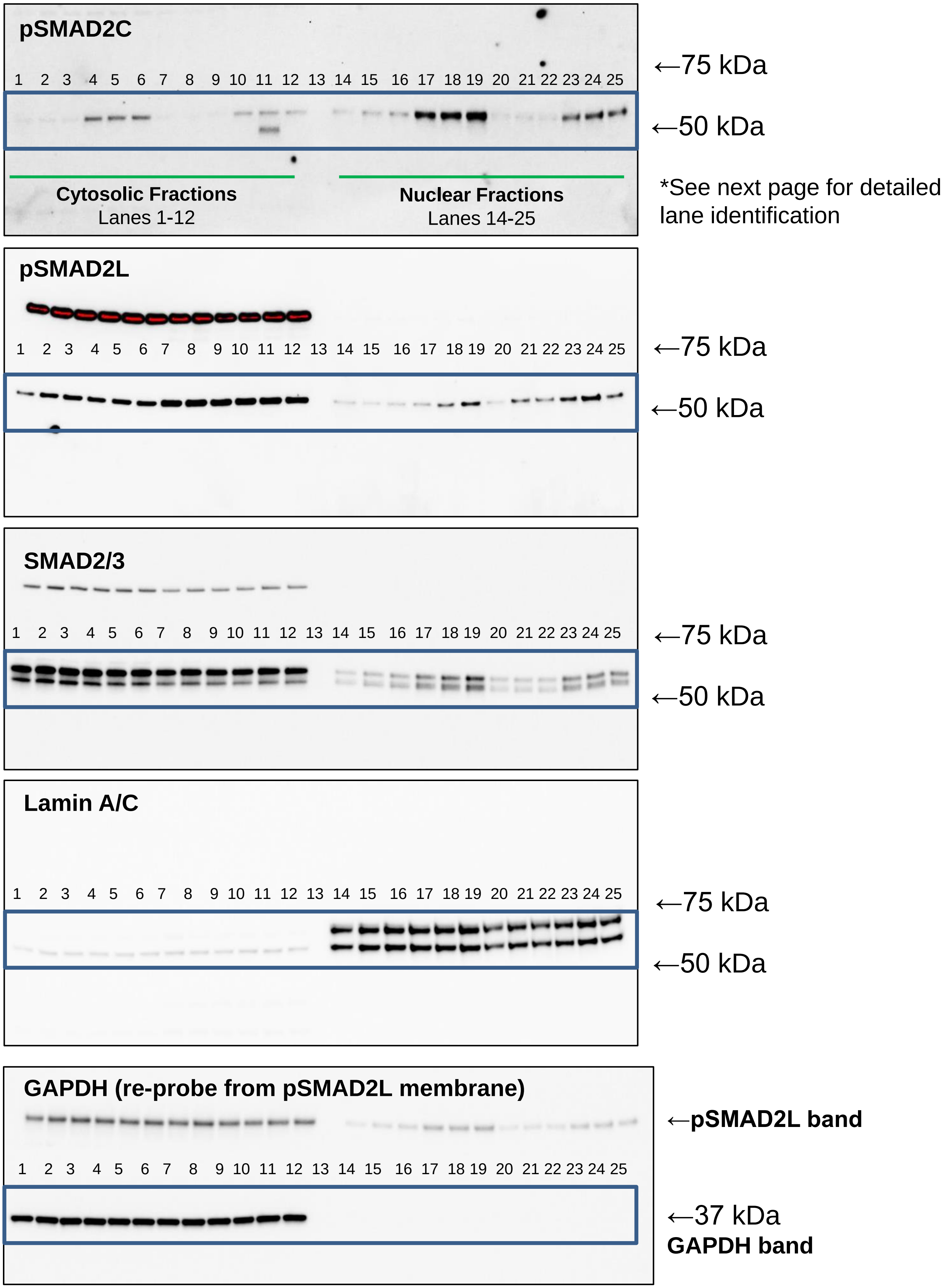

Uncropped Blots from Figure 5C (continued)

Active JNK + Myostatin Experiment in C2C12
Cytosolic Fractions
Lane
1-3: EV + Vehicle Control
4-6: EV + 20 nM Myostatin
7-9: Active JNK + Vehicle Control
10-12: Active JNK + 20 nM Myostatin
13: Empty Lane
Nuclear Fractions
Lane
14-16: EV + Vehicle Control
17-19: EV + 20 nM Myostatin
20-22: Active JNK + Vehicle Control
23-25: Active JNK + 20 nM Myostatin

Uncropped Blots from Figure 7A

*See next page for detailed lane identification

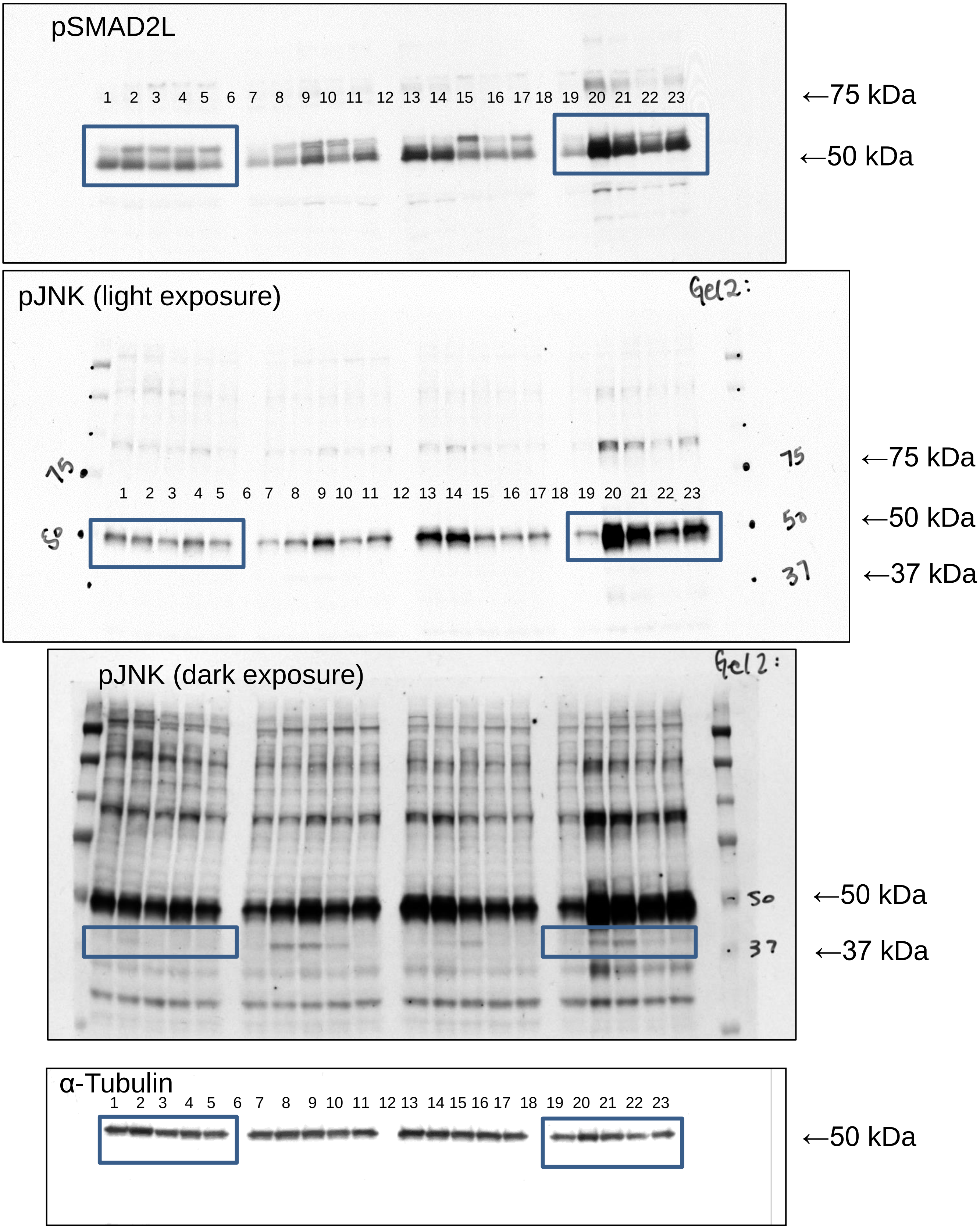

Uncropped Blots from Figure 7A (continued)

Human Endurance and Resistance Exercise Time-Course Samples

Lane:
1: Subject C3 Rest
2: Subject C3 0 min Post-Exercise
3: Subject C3 15 min Post-Exercise
4: Subject C3 30 min Post-Exercise
5: Subject C3 60 min Post-Exercise
6: Empty Lane
7: Subject R3 Rest
8: Subject R3 0 min Post-Exercise
9: Subject R3 15 min Post-Exercise
10: Subject R3 30 min Post-Exercise
11: Subject R3 60 min Post-Exercise
12: Empty Lane
13: Subject C4 Rest
14: Subject C4 15 min Post-Exercise
15: Subject C4 0 min Post-Exercise
16: Subject C4 30 min Post-Exercise
17: Subject C4 60 min Post Exercise
18: Empty Lane
19: Subject R4 Rest
20: Subject R4 0 min Post-Exercise
21: Subject R4 15 min Post-Exercise
22: Subject R4 30 min Post-Exercise
23: Subject R4 60 min Post-Exercise
